# Round the Hospitals

**Published:** 1919-03-15

**Authors:** 


### ROUND THE HOSPITALS.

The appointment of Miss Monk to be successor to Miss Luckes in the office of matron of the London Hospital was a foregone conclusion. She has worked under her great chief as principal matron's assistant for over seven years, has often held the reins in Miss Luckes' absence. Miss Monk was appointed acting matron for about six months, and filled the position with great ability. There will thus be no interregnum and no period of indecision and suspense such as often attends the break up of a long and distinctive term of office. These facts do not preclude the possibility that as time goes on Miss Monk may introduce changes, for however harmoniously two

**ROUND THE HOSPITALS—**(*continued*).

women work together, however loyally the one may subordinate her views to those of the other, it is certain that new responsibility brings with it a new outlook. Progress on somewhat newer lines is entirely consistent with reverence for a chief whose work is accomplished. Miss Monk was awarded the Royal Red Cross, Second Class, towards the end of 1917. She began her training at the Hospital for Women, Liverpool, and entered as a probationer at the London Hospital in 1904. On the completion of her period of training, she held various nursing appointments in the hospital, and was appointed a Sister in the Preliminary Training-School at Tredegar House.

The new matron is distinguished among her many gifts by a more complete training in commissariat matters than has often fallen to the lot of any nurse. Owing to her housekeeping talents she was appointed steward of the London Hospital at the beginning of 1918, and thus has had splendid opportunities of mastering the many intricate matters involved in buying, storing, distributing, cooking, and serving the hospital food supplies. She carried out her duties with great success. Not only was she responsible for, and became expert in the purchase of such commodities as milk, bread, groceries, etc., but she had control over a porter staff of more than ninety men. Her organising ability showed itself in many ways, and especially in her endeavour to shorten the porters' long hours, without detriment to the work of the hospital. Exactly one year later she was, by the unanimous wish of the House Committee, appointed matron. If there were only leisure in her life for writing a book on her experiences what points of vital interest to matrons and managers might she not unfold! Her administrative talents have been of great value in the department for the treatment of wounded officers, which she directed. May her election bring many essential changes and enhanced benefits to all London nurses.

An impressive tribute was paid to the memory of Miss Luckes at the annual meeting of the King Edward VII. Hospital, Cardiff, by Colonel Bruce Vaughan, and a motion of sympathy with Lord Knutsford, chairman, and the members of the Board of Management of the London Hospital was carried in silence, all standing. Colonel Bruce Vaughan recalled how ready Miss Luckes had ever been to help them with advice and encouragement. Quite recently she had complimented them on the foundation of a Probationers' Training School, and he would like to state there that it was to her foresight and keen interest in the theoretical training of nurses that five preliminary schools were established in this country.

Following on the appointment of Miss M. Vaughan-Winters as sister tutor at the General Hospital, Nottingham, which we reported last November, a Preliminary School for Probationers on somewhat novel lines has been started at this institution. Five pupils (of whom one proved unsuitable) entered for the course on January 1, and have now, after eight weeks' work, successfully passed a qualifying examination in which an average of 80 out of 100 possible marks was attained. A well-thought-out scheme of theoretical instruction was supplemented by practical work in the wards under the instruction of the tutor sister, arrangements being made with the ward sister to reserve the special work for the week. During the course the pupils were initiated, from 7 to 10 A.M., into the best way of keeping clean the milk larder, pantries, ward kitchens, and sanitary blocks. Alternate mornings and afternoons were given up to recreation and study. As far as possible theory and practice went hand in hand. Towards the end of the course practical nursing was taught, demonstrations were given, and when possible the work was actually done by the pupils. The scheme may suit moderate-sized hospitals, and meet with imitation. The experiment reflects credit on the matron, Miss Kendall, and the sister tutor.

A great forward step has been taken at the Royal Infirmary, Preston, in response to representations from the matron, Miss Marks. The board of management has unanimously agreed to give the nursing staff one day off in seven as soon as the necessary building is completed to accommodate the additional nurses. Additions to salaries have also been made. All the sisters are to have an extra £5 a year. Probationers' salaries have been raised to £16, £18, £20, £30, with uniform. The nurse who gains highest marks in her final examination is to have free midwifery training, with preparation for the C.M.B. certificate during her fourth year. The private nursing staff are to receive a bonus of 5 per cent. in the first year and 10 per cent. in subsequent years on all their earnings, in addition to their salary of £40 a year. We hail with the greatest satisfaction the inclusion of this important Lancashire institution among the little band of pioneers in the "one day in seven" movement.

The pay of probationers at the Dreadnought Hospital, Greenwich, has been revised by the committee, and salaries have been considerably augmented. In future probationers will receive £20 in their first year, in the second year £22, and in the third year £25. Candidates are directed to apply to the Matron at the Seamen's Hospital, Greenwich.

An additional sum of £7,000 a year is required by the Queen Victoria Jubilee Institute for Nurses to meet the increased cost of the training, supply, and supervision of nurses, to meet an anticipated reduction of fees to be received from the Association, to enable them to increase the salaries of their nurses, and to add to the Queen's Nurses' Benefit Fund. The Duke of Portland, presiding on March 4 at a meeting of the Queen's Fund, brought the new needs of the Jubilee Institute forcibly before a distinguished gathering. He recalled the fact that in August last as an emergency measure the minimum clear salary for a Queen's nurse was increased to

**ROUND THE HOSPITALS—*(continued).***

£40 but the Council thought every Queen's nurse ought to have at least £50. It seemed to him an obligation of honour upon nursing associations to see that the nurses received adequate remuneration, and he thought many of the nurses and staff of the Institute ought to receive higher salaries than at present, including inspectors and nurses in training for district work. In order to help the associations the Institute has lowered the former fee of £4 per nurse per annum charged for supplying Queen's nurses to an affiliation fee of one guinea per annum.

A progressive rise of salaries has been in force since war began in the Forest Hospital within the borough of Mansfield, which illustrates better than any example we have yet seen the upward trend of nurses' pay. The hospital is a small one with but thirty-two beds, and, since it is in small hospitals that unsatisfactory conditions of work and pay are apt to be perpetuated long after they have been banished from large establishments, the history of the last few years is the more remarkable. Pre-war salaries were: Matron, £65; sister, £35, staff nurse, £28 to £32; probationers, £10, £14. In 1916 salaries were raised to: Matron, £72; sister, £40; staff nurse, £30; probationers, £14, £15. In 1917, in lieu of war bonus, the committee raised salaries again to matron, £90; sister, £45; staff nurse, £35; probationers, £18, £25. Last year the committee decided to make a further advance. The matron now receives £100; the sister, £55; staff nurse, if with general training £45, if fever certificated only £35; probationers, £20, £25. In all cases uniform ready to wear is given. Each nurse and maid has her own room. Off-duty time is 2½ hours daily, 4½ on Sunday, half a day once a week, a whole day once a month, with a long week-end at Christmas.

Cardiff has been going through a very troubled time owing to a renewal of the influenza outbreak. At King Edward VII. Hospital they have had to close down the out-patient department owing to shortage of nurses. Many of the nursing staff have taken the infection, and in the absence of a private staff the hospital has been unable to meet any of the urgent calls for assistance with which it has been besieged. A ward released from military occupation has been fitted up for pneumonia cases, although it is feared this may prove a great strain on the nurses. The city sanatorium for infectious cases is entirely inadequate, and has applications for thirty-seven more cases than it has beds. The hospital authorities are a little sore at the fact that up to now not a single unmarried nurse has been demobilised for service at the hospital.

The Hertfordshire County Council has already distinguished itself by its able memorandum on district nurses' pay, which has had an incalculable influence for good. The keen sense displayed by its members of the importance of good nursing, and their perception that just pay is essential if the standard of women taking up district work is to be maintained, prove the existence of some sound thinkers on the Council. This is further evidenced by the schemes which the Council now has in hand. There are to be good cottages built for the police and the roadmen within its boundaries. There is to be a County Council sanatorium to accommodate some 100 patients. There are to be maternity, child-welfare, and school clinics. A convalescent home is projected near the sea for children and nursing mothers, and there is to be a large residential home for mentally defective children. This is a goodly instalment of reconstruction. The Council understands the value of good work, and should be able to enlist the services of some first-rate nurses to help in carrying out these enterprises.

Every trained nurse who has intelligence and the power of thought and observation must have been struck with the abundant evidence afforded of the unwarrantable methods which some enemies of the College have resorted to during the last few months. Amongst them may be noted the proceedings at the meeting at the Royal Society of Medicine in January, the disregard of the ancient and universal rule in regard to the dead, and the attempt to trap the College and capture its work for nurses, which are but a few of the evidences of the methods of enemies of the College which all nurses should note and resent. The College throughout has worked steadily under great difficulties to help the nurses to help themselves, a fact which every capable nurse should realise and rejoice at. Then she will surely rally to the support of the College and lose no opportunity to recruit trained nurses to join the College Register without delay. Our readers will be glad to learn that the Salaries and Conditions of Employment Committee appointed by the College of Nursing (Limited by Guarantee) is obtaining useful statistical evidence covering a wide area of all branches of the nursing profession. Nurses will be given an opportunity of placing evidence before the Committee as its work proceeds, and due notice to this effect will shortly be issued. Meanwhile we invite all who have points they are desirous of raising to forward us particulars, which we will in due course hand to the College authorities to be dealt with. It is satisfactory to add that the work of the College in regard to all questions affecting the salaries and conditions of employment of trained nurses is proceeding continuously and successfully at present. Much energy is being put into the work, and the results anticipated promise to prove of great importance.

The activities of the College of Nursing and its influence for good are well illustrated by the Council's action in regard to the pay of nurses in the Royal Naval Nursing Service. A correspondence has taken place between the Admiralty authorities and the College Council which has resulted in an intimation from the former, dated 4th March, that the question of an increase of salary to members of the Queen Alexandra Royal Naval Nursing Service is now under consideration, and it is hoped that a scheme for improvement in the scale of pay

**ROUND THE HOSPITALS—(*continued*).**

allowed to these ladies will be approved in the near future. Such a result is very gratifying. It should encourage trained nurses to join the College Register quickly, and to send any question which may arise that affects their interest to the Secretary of the College of Nursing, 7 Henrietta Street, W.1. inviting the help of the College Council.

The King has been pleased to award the Royal Red Cross to the following ladies of the Nursing Services in recognition of their valuable services with the Forces in Mesopotamia:—First Class: Sister E. H. M. Downie, Sister D. Rothery, and Sister E. C. Sutton, A.R.R.C., Q.A.I.M.N.S.R.; Sister N. Nicholls, T.F.N.S. Second Class: Sister M. A. Brown, Staff Nurse C. E. Eason, Staff Nurse M. A. Edge, Sister C. M. Henderson, Staff Nurse M. A. Jones, Sister B. F. Joyce, Staff Nurse A. M. Letts, Sister J. Mann, Sister E. A. Morris, Sister A. W. Nice, Staff Nurse A. Osborne, Sister I. C. McL. Young, all of Q.A.I.M.N.S.R.; Mrs. A. M. George, T./Nurse, Temp. Nurs. Serv., India; Miss F. M. Mendes, T./Nurse, Temp. Nurs. Serv.; Sister E. Swift, T.F.N.S.

The King held an Investiture in the Ballroom of Buckingham Palace on March 8 at 11 a.m. His Majesty personally bestowed the Royal Red Cross (Second Class), on the following members of the Nursing Service:—Sister Elizabeth Lawton and Staff Nurse Elizabeth Campbell, Q.A.I.M.N.S.R.; Sister Frances Brown, B.R.C.S.; Staff Nurse Alice England, Royal Naval Hospitals. Matron Constance Todd, St. John's Ambulance Brigade, had the honour of receiving the Military Medal for bravery in the field from the King.

Best congratulations to Brighton on its new Centre of the College. There is hardly any place on the South Coast more frequented by private nurses, and the Centre will be a real source of encouragement to members of this branch of the profession who are often singularly lonely. They, more than any institution nurses, stand to profit by post-graduate lectures, and it is they who most need that quickening of vocation and electric current of professional stimulus which ensue. The Centre will find a great source of strength in the matron, sisters, and nurses of the Royal Sussex County Hospital.

In both Manchester and Liverpool grave difficulty is being experienced in nursing influenza patients, and in Liverpool cautions have been issued that persons attending influenza should wear masks. At Manchester they are trying to organise a body of volunteer nurses. Patients' visitors are being excluded during the epidemic from the Manchester Royal Infirmary. At Newcastle, where influenza has been raging, it appears to be rapidly abating, and it is hoped the worst is over.

At the Nurses' Re-Settlement Bureau, 16 Curzon Street, Mayfair, our representative learns that about 3,000 nurses, trained and untrained, have been demobilised since the beginning of the year. Some of the V.A.D.s are turning to midwifery and dispensing, some of the trained nurses to medicine and surgery. There is a great demand just now for private nurses, twenty applications for them having been received in one week. Considerable difficulty has been experienced by nurses in filling up the Civil Employment form, and, although we are aware that accurate filling in of forms is not exactly a strong point in the nursing profession, we would like to say, in their defence, that the form provided for the use of demobilised nurses is one of the most soul-destroying we have ever had brought to our notice. Including sub-headings, it asks for no fewer than forty-three separate items of information.

At the annual meeting of the Kingston Nursing Association a very satisfactory financial statement was read, from which it appeared that the excess of income over expenditure was £24, and that as compared with the previous year nearly every item in the receipts showed an increase and most of the items of expenditure a decrease—a tribute to the economical management of the lady superintendent. Miss McClymont resigned her post in May last, and was much regretted by the committee. Her very capable successor is Miss Harborough. The nurses appear to be very popular, and two of them, Nurse Davidson and Nurse Pearce, are alluded to by the committee as their old friends, well known and much loved in the district.

The inquest on the body of Jessie Charlotte Spurling, who died in the nursing home known as Wittring Court, near Leigh-on-Sea, was concluded on March 5. The jury returned a verdict that the death of Miss Spurling was caused by neglect and starvation, and that the persons responsible were Mrs. D. A. Binstead and Mr. H. Hall. This is tantamount to a verdict of manslaughter. Mrs. Binstead appears to be suffering from brain disease, is partially paralysed, and subject to fits of great mental excitement. The Coroner said he had never seen anything to equal the terrible filth at Wittring Court. He blamed those who had been going in and out for not reporting the terrible condition of the "home," and expressed his astonishment that the rural council, notified by Dr. Adams of the state of affairs in December, had taken no steps until after the death of Miss Spurling. He considered Mr. Hall must have known something of what was going on. As a result of the inquiry the Coroner hopes to see the law so amended or altered that no person can take in for gain or otherwise any person of this character unless they are subject to proper supervision by some Government department. The case painfully illustrates the reluctance of individuals to step forward and expose abuses, however flagrant, by which they are not personally inconvenienced.

